# The Development of a Quantitative Disability Assessment Tool in Patients with Idiopathic Parkinson’s Disease

**DOI:** 10.3390/diagnostics14182063

**Published:** 2024-09-18

**Authors:** Han Gil Seo, Seo Jung Yun, Yoojin Song, Ho Seok Lee, Dae Hyun Kim, Won Hyuk Chang

**Affiliations:** 1 Department of Rehabilitation Medicine, Seoul National University College of Medicine, Seoul National University Hospital, Seoul 03080, Republic of Korea; hangilseo@snu.ac.kr (H.G.S.); seojungyun@snu.ac.kr (S.J.Y.); 2 Department of Physical and Rehabilitation Medicine, Center for Prevention and Rehabilitation, Heart Vascular and Stroke Institute, Samsung Medical Center, Sungkyunkwan University School of Medicine, Seoul 06351, Republic of Korea; syoojin1228@gmail.com (Y.S.); hoseok89.lee@samsung.com (H.S.L.); dh23.kim@samsung.com (D.H.K.); 3 Department of Health Sciences and Technology, Samsung Advanced Institute for Health Science and Technology, Sungkyunkwan University, Seoul 06355, Republic of Korea

**Keywords:** Parkinson’s disease, disability, activities of daily living, quality of life, validation

## Abstract

**Background/Objectives:** The objective of this study was to develop a novel quantitative disability assessment tool for patients with idiopathic Parkinson’s disease (IPD). **Methods:** A total of 47 patients with IPD were recruited from two hospitals. A specialist in Rehabilitation Medicine utilized the modified Schwab and England Activities of Daily Living Scale (mSEADL) as a reference, conducting a comprehensive medical chart review and an in-depth interview. The novel-developed disability measurement was calculated as ((mSEADL during the on-state) × (time of on-state)) + ((mSEADL during the off-state) × (time of off-state))/(waking time). Additionally, the degree of disability was assessed using the Korean version of the Modified Barthel Index during the on-state. **Results:** Twenty-four participants (51.1%) exhibited the off-state during waking hours. In patients exhibiting an off-state, the mSEADL score was significantly lower during the off-state than during the on-state (*p* < 0.05). The novel-developed disability measurement demonstrated a higher correlation coefficient with the reference mSEADL (0.960) than with the K-MBI (0.808), with statistical significance (*p* < 0.05). **Conclusions:** The results demonstrated that larger IPD patients exhibited an on–off phenomenon with greater dependency during the off-state. Therefore, the on–off phenomenon should be considered when evaluating disability in patients with IPD, with methods such as the novel-developed disability measurement tool in this study.

## 1. Introduction

Idiopathic Parkinson’s disease (IPD) is a neurodegenerative disease that affects the motor system and has a direct impact on motor control and mobility in general [1]. In the absence of a disease-modifying therapy for IPD patients, the progressive nature of the disease will inevitably result in a decline in the patient’s independence and quality of life (QoL) [2]. Consequently, it is of great importance to accurately assess and maintain the patient’s independence throughout the rehabilitation process. The Unified Parkinson’s Disease Rating Scale (UPDRS) motor section (part III) [3] and the modified Hoehn and Yahr (mH&Y) scale [4] are the most commonly utilized instruments for rating the disease stage. Although the mH&Y scale is the most commonly utilized assessment for evaluating loss of independence in IPD patients [5], it is important to note that the mH&Y scale is a tool designed to quantify motor impairment, not to assess independence. Moreover, the majority of IPD patients also present with non-motor symptoms, including cognitive impairment, insomnia, depression and anxiety, bladder dysfunction, pain, and fatigue [6]. These non-motor symptoms, in addition to motor symptoms, have the potential to influence disability, loss of independence, and QoL in IPD patients [6]. Non-motor symptoms have also been identified as a significant contributor to the caregiver’s burden [7]. In particular, cognitive impairment has been shown to have a particularly detrimental impact on the caregiver’s burden for IPD patients [8]. The Schwab and England ADL Scale (SEADL) has also been employed as a disease-specific disability measurement tool [1]. The generic 10-item Barthel ADL Index has also been utilized for the measurement of disability in IPD patients [5,9]. However, all three scales, i.e., the mH&Y scale, SEADL, and Barthel ADL Index, have been demonstrated to lack the capacity to serve as an appropriate instrument for the assessment of dependency in basic ADL in IPD patients [10].

The wearing-off phenomenon may be attributed to the lack of a satisfactory tool for assessing dependency in IPD patients. IPD patients frequently experience the wearing-off phenomenon as the disease progresses, and it has been demonstrated to significantly reduce disability and QoL [11]. Many IPD patients with the wearing-off phenomenon may show more disability during the off-state as drug treatment is less effective. Therefore, it is not feasible to accurately assess the extent of disability within the limited time frame of a medical visit for IPD patients. The difficulty in assessing the degree of disability in IPD patients has been previously reported; it is most appropriate to evaluate the degree of disability not by a single measurement tool but by a specialist who has sufficient clinical experience [10]. However, the evaluation of the degree of disability by specialists has the disadvantage of not being able to be widely used clinically. Therefore, it is necessary to evaluate the degree of disability concerning the on–off phenomenon in IPD patients.

Despite the existence of a number of assessment tools designed to quantify disability in IPD patients, no tool has yet considered the on–off phenomenon. This study aimed to develop a novel quantitative disability assessment tool for use in patients with IPD.

## 2. Materials and Methods

### 2.1. Study Design and Participants

IPD patients were enrolled from two disparate medical facilities. The inclusion criteria were as follows: (a) patients who had been diagnosed with IPD, (b) aged over 19 years, and (c) who had completed the structural questionnaire without exhibiting definitive cognitive impairment (Korean Mini-Mental State Examination [K-MMSE] > 19) [12]. An IPD diagnosis was made on the basis of medical history, physical examination, and neuroimaging studies [13]. Patients were excluded if they had a preexisting and active major neurological disease or a preexisting and active major psychiatric disease, such as major depression, schizophrenia, bipolar disorder, or dementia. The Institutional Review Board (IRB) of each hospital approved this study protocol (IRB of Samsung Medical Center (IRB no. 2019-07-025) and IRB of Seoul National University Hospital (IRB no. 1907-193-1052)), and informed consent was obtained prior to participation.

### 2.2. Disability Assessments

In this study, the disability of each participant was assessed using three different measurement tools that are currently in use. The initial assessment tool was the mSEADL, a disease-specific measurement tool for disability, which ranges from 100% (completely independent, essentially normal) to 0% (bedridden, vegetative function, completely invalid) [1]. As the mSEADL is regarded as the most effective method for assessing the extent of disability in IPD patients, this tool was employed as the reference value for disability in this study (HGS and WHC in movement disorders) [10]. Each physiatrist employed the mSEADL to assess the degree of disability through a comprehensive medical chart review and an in-depth interview. Additionally, experienced researchers (YS and SJY) evaluated a distinct disability measurement tool, the Korean Version of the Modified Barthel Index (K-MBI), in the context of rehabilitation outpatient clinics for IPD patients. The K-MBI ranges from 0 to 100, with higher scores indicating greater ADL independence [14].

The novel-developed disability measurement in this study was calculated as follows:

$$\frac{\begin{array}{c} \left(\mathrm{mSEADL}\;\mathrm{during}\;\mathrm{on}-\mathrm{state}\right)\times \left(\mathrm{time}\;\mathrm{of}\;\mathrm{on}-\mathrm{state}\right))+(\left(\mathrm{mSEADL}\;\mathrm{during}\;\mathrm{off}-\mathrm{state}\right)\times \left(\mathrm{time}\;\mathrm{of}\;\mathrm{off}-\mathrm{state}\right) \end{array}}{\mathrm{Waking}-\mathrm{Time}}$$

Each mSEADL was assessed by an experienced researcher (YS or SJY) during both the on-state and off-state periods. Waking time was defined as the sum of time spent in both the on-state and off-state periods. All assessments were conducted by experienced researchers (YS and SJY) without knowledge of the results provided by physiatrists. The novel disability measurement was calculated using mSEADL, with higher scores indicating greater independence in activities of daily living (ADL).

### 2.3. Other Assessments

A variety of clinical and demographic variables were assessed, including age, sex, disease duration, and disease status, with the use of the mH&Y scale for the assessment of the severity of IPD [4]. The Geriatric Depression Scale—Short Form (GDS-SF) [15] was employed to assess mood, and the K-MMSE [12] was utilized to evaluate cognitive function. The GDS-SF comprises 15 yes/no questions and is a self-report measure of depressive symptoms. All participants were queried as to whether they had experienced any of the aforementioned symptoms over the previous week. The maximum score that can be achieved on the GDS-SF is 15, with higher scores indicating a greater severity of symptoms. Additionally, the UPDRS was employed for the purpose of measuring the clinical characteristics and motor function [16]. The UPDRS comprises four distinct sections. Part I (mentation, behavior, with mood scores ranging from 0 to 16), Part II (activities of daily living with scores ranging from 0 to 52), Part III (motor examination with scores ranging from 0 to 108), and Part IV (complications, with scores ranging from 0 to 23) [3]. In this study, the UPDRS was administered in its entirety, comprising Parts I, II, and III. Furthermore, the Parkinson’s Disease Questionnaire-39 (PDQ-39), a disease-specific QoL measure, was employed to ascertain the QoL [17]. The total score is converted into the PDQ-39 Summary Index (PDQ-39 SI), which ranges from 0 to 100 points. A lower score indicates a higher quality of life [18]. All functional examinations and surveys were conducted by experienced researchers in the field of movement disorders (YS and SJY).

### 2.4. Statistical Analysis

The statistical analyses were conducted using SPSS version 27.0 (SPSS, Chicago, IL, USA). The Shapiro–Wilk test was employed to ascertain the distributional normality of all continuous variables. Given that some continuous variables were not found to be normally distributed (*p* < 0.05), non-parametric statistical analyses, including the Mann–Whitney U test and Spearman’s correlation analysis, were performed. The IPD patients were initially divided into two groups: those exhibiting the on–off phenomenon and those not displaying this phenomenon. The clinical and functional characteristics of IPD patients were compared between the two groups. A correlation analysis was conducted to assess the reliability of each disability measurement with the reference value of the disability in each total patient, as well as in patients with and without the off-state. The strength of the relationship was interpreted as perfect (+1.0), very strong (1.0~0.8), moderate (0.6~0.8), fair (0.3~0.6), poor (0.1~0.2), or none (0.0) by Spearman’s correlation coefficients [19]. *p*-values less than 0.05 were considered statistically significant.

## 3. Results

Table 1 presents the demographic and clinical characteristics of the 47 IPD patients who participated in this study. The mean age (range) of the patients was 68.2 years (51–85 years), 59.6% (*n* = 28) were male, and the mean disease duration (range) was 6.7 years (0.9–22.1 years).

### 3.1. Comparison between IPD Patients with and without the Off-State

Of the 47 patients, 24 (51.1%) reported the on–off phenomenon. No significant differences were observed in age, sex, disease duration, mH&Y scale, or UPDRS Part III between patients with and without the off-state. However, there were significantly higher UPDRS Part I and Part III scores in patients with the off-state than in patients without the off-state (*p* < 0.05). Additionally, each GDS-SF and PDQ-39 SI scores were significantly higher in patients with the off-state than in patients without the off-state (*p* < 0.05, Table 1).

The duration of waking time did not differ according to the on–off phenomenon. However, in patients with the off-state, an off-state occurred for an average of 3.9 h (23.1%) during waking time. No significant difference was observed in the mSEADL during the on-state between patients with and without the off-state (Table 2). In patients exhibiting an off-state, the mSEADL score during the off-state was significantly lower than that during the on-state (*p* < 0.05). The calculated mSEADL score, representing a novel disability measurement, was 76.5 and 71.5 in patients with and without the off-state, respectively. The reference mSEADL score was 76.7 and 71.3 in the patients with and without the off-state, respectively. The calculated mSEADL score and the reference mSEADL score were significantly lower in the patients with the off-state than in those without (*p* < 0.05). However, there was no significant difference in K-MBI according to the on–off phenomenon (Table 2).

### 3.2. Correlation Analysis with the Calculated Disability Measurement

Table 3 shows the correlation coefficient between the novel-developed disability measurement and other disability measurements. The novel disability measurement demonstrated a statistically significant correlation with the reference mSEADL, K-MBI, and UPDRS Part II in all IPD patients, as well as in patients with and without the off-state, respectively (*p* < 0.05). The correlation between the novel disability measurement and the reference mSEADL was particularly strong in all IPD patients, as well as in patients with and without the off-state, respectively. However, the correlation between the novel disability measurement and the other disability measurements was moderate in all IPD patients, as well as in patients with and without the off-state.

## 4. Discussion

This study demonstrates that the on–off phenomenon occurred in a considerable number of IPD patients, and the issue of functional evaluation during the on-state was identified. The novel disability measurement tool developed in this study was demonstrated to be a quantitative methodology for evaluating disability in IPD patients.

It is known that a considerable proportion of IPD patients exhibit an on–off phenomenon [20,21], which is associated with lower depression and QoL [22]. In this study, 51.1% of IPD patients with the on–off phenomenon showed more depressive mood and lower QoL compared to those without the on–off phenomenon. This finding is in accordance with the results of previous studies [20,21,22]. However, no significant differences were observed between the two groups with regard to the UPDRS Part III, the mH&Y scale, and the K-MBI administered by raters in the hospital. This discrepancy may be attributed to the observation that the majority of IPD patients exhibiting the on–off phenomenon are in the on-state during periods of activity, particularly when receiving medical care at a healthcare facility [23]. Therefore, the assessment of disability by hospital-based raters might not fully reflect the characteristics of IPD with fluctuating symptoms, which could potentially compromise the accuracy of disability assessment [24]. Accordingly, a novel assessment approach is required that captures the characteristics of IPD, including the on–off phenomenon. In this study, we developed a novel approach to disability evaluation, which involves calculating the time spent in an off-state and the degree of impairment from the time spent awake. A comparison with a disability measurement reflecting IPD characteristics, as determined by experts in the disability assessment of Parkinson’s disease patients, revealed a highly significant correlation. In particular, the novel disability measurement was found to be highly correlated with all IPD patients, regardless of the on–off phenomenon. Furthermore, the novel disability measurement is relatively simple to evaluate, which may facilitate its implementation in the disability assessment of IPD patients in the future.

Tools to assess the degree of disability in Parkinson’s disease patients are divided into generic measures and disease-specific measures [24]. Generic measures are tools designed for use in healthcare planning, general health surveys, and comparisons with other disease groups or the general population. One such measure is the K-MBI, which is not well suited to cases of fluctuating function, such as Parkinson’s disease patients with the on–off phenomenon. This is because the K-MBI requires the examiner to evaluate and measure the actions performed by the subject [14]. The results of this study indicate that the K-MBI does not accurately reflect the characteristics of patients in the off-state. Furthermore, the analysis revealed no statistically significant difference between patients with and without the off-state. While there was a notable correlation between this novel disability measurement and the K-MBI, the K-MBI is an inadequate standalone assessment tool for disability in IPD patients. Conversely, as a disease-specific measure of IPD, it is challenging to make comparisons with patients suffering from other diseases. However, it is a tool designed to ascertain disease-specific issues and the impact of treatment. In this study, we employed UPDRS Part II in conjunction with the most widely utilized mSEADL. In contrast to the K-MBI, UPDRS Part II is more suitable for assessing disability in IPD patients than the K-MBI, given that IPD patients in an off-state are rated as more impaired than those without an off-state. Additionally, the robust correlation between the ADL subscore and disease duration in PD indicates that this metric may offer a more accurate representation of disease progression than the signs and symptoms assessed in other UPDRS subscores [25]. However, a number of issues with the original scale were identified, leading to the development of the MDS-UPDRS. This new scale was designed to assess a wider range of aspects of Parkinson’s disease, including non-motor and motor activities of daily living, as well as motor complications. Unlike UPDRS Part II, the MDS-UPDRS does not directly assess ADLs, which is why UPDRS Part II is not widely used to assess disability in IPD [26]. mSEADL is designed to foster independence in practical, real-life activities, with an emphasis on movement at varying levels of function, distinguished by degree or nature of slowness. This approach has the potential to be beneficial in the treatment of motor system disorders. Moreover, the method can be applied to non-motor disorders or dysfunction, as slowing can be indicative of disability resulting from primary cognitive as well as movement disorders [27,28]. In this study, we developed a novel disability measurement using a new quantitative formula based on the mSEADL and compared it with the most widely recommended disability measurement by a specialist with sufficient clinical experience [10]. The novel-developed disability measurement in this study exhibits a markedly strong correlation with disability measurement by a specialist with substantial clinical experience. It is anticipated that this novel approach will facilitate more precise assessments of IPD patient functioning, even by non-expert clinical research personnel or care staff members conducting patient interviews.

There are several limitations in this study. Firstly, the number of patients was relatively small, and they were recruited from a tertiary hospital, which may limit the generalizability of the findings to all IPD patients. A second limitation of this study is that not all disabilities could be analyzed according to the mH&Y scale. Although this study included patients with a relatively wide range of mH&Y scales, further research is required to ascertain the relevance of the novel disability measurement for different mH&Y scales. Further research with a larger patient cohort will be required to substantiate these findings. Furthermore, as the subjects were only assessed at a single point in time, it is not possible to determine whether the novel disability measurement can reflect the degree of functional change as the disease progresses. In particular, disability in IPD patients tends to worsen as the disease progresses. Consequently, further research is required to ascertain the implications of the novel-developed disability measurement proposed in this study over time in the same patient population. This study is insufficient to support the use of the novel disability measurement as an intervention outcome in IPD patients, and further research will be necessary. Moreover, the care of IPD patients should encompass not only the disability but also the QoL of these patients. Further research is required to ascertain whether the novel-developed disability measurement in this study is associated with QoL in IPD patients. It is established that difficulties in facial emotion recognition, such as those encountered with masked faces, also impact the QoL in IPD patients [29,30]. This should be taken into account in the development of future tools for assessing QoL in patients with Parkinson’s disease.

In spite of these several limitations, the results in this study demonstrated that a relatively larger number of IPD patients showed the on–off phenomenon with more dependency during the off-state. IPD patients with the on–off phenomenon showed a significantly higher depressive mood and lower QoL than PD patients without the on–off phenomenon. Therefore, the on–off phenomenon should be considered to evaluate the disability in IPD patients such as the novel-developed disability measurement in this study.

## Figures and Tables

**Table 1:** Characteristics of participants.

	Total Patients (*n* = 47)	According to the On–Off Phenomenon		
		Patients with the Off-State (*n* = 24)	Patients without the Off-State (*n* = 23)	*p*-Value
Age (mean ± SD, y)	68.2 ± 7.9	67.5 ± 7.7	69.0 ± 8.1	0.733
Sex (M:F)	28:19	16:8	12:11	0.312
Disease duration (mean ± SD, y)	6.7 ± 4.8	8.3 ± 5.7	5.0 ± 3.0	0.066
Modified Hoehn and Yahr stage (1:1:5:2:2.5:3:4:5)	6:4:8:10:9:8:2	1:1:7:5:4:5:1	5:3:1:5:5:3:1	0.188
UPDRS Part I	2.8 ± 2.4	3.4 ± 2.5	2.2 ± 2.1 *	0.044
UPDRS Part II	13.2 ± 9.5	15.9 ± 8.8	10.4 ± 9.6 *	0.017
UPDRS Part III	25.3 ± 15.6	27.2 ± 17.5	23.3 ± 13.5	0.551
K-MMSE	27.6 ± 2.1	28.3 ± 2.1	26.8 ± 1.9 *	0.007
GDS-SF	7.6 ± 2.7	8.9 ± 2.6	6.3 ± 2.2 *	<0.001
PDQ-39 Summary Index	27.7 ± 18.7	37.4 ± 16.4	17.5 ± 15.4 *	<0.001

UPDRS, Unified Parkinson’s Disease Rating Scale; K-MMSE, Korean Mini-Mental State Examination; GDS-SF, Geriatric Depression Scale—Short Form; PDQ-39, Parkinson’s Disease Questionnaire-39. * *p* < 0.05, compared with the patients with the off-state.

**Table 2:** Comparison in disability measurements according to the on–off phenomenon.

	Patients with the Off-State (*n* = 24)	Patients without the Off-State (*n* = 23)	*p*-Value
Waking time (mean ± SD, h)	16.9 ± 2.0	16.9 ± 1.3	0.974
On-state (mean ± SD, h)	12.8 ± 3.7	16.9 ± 1.3 *	<0.001
Off-state (mean ± SD, h)	3.9 ± 2.6	-	-
mSEADL during the on-state (mean ± SD)	74.6 ± 18.2	76.5 ± 19.0	0.508
mSEADL during the off-state (mean ± SD)	64.2 ± 21.9 ^†^	-	-
Novel-developed disability measurement (mean ± SD)	71.5 ± 20.3	76.5 ± 19.0 *	0.048
Reference mSEADL (mean ± SD)	71.3 ± 20.9	76.7 ± 19.0 *	0.049
K-MBI (mean ± SD)	87.2 ± 16.1	88.9 ± 18.1	0.265

mSEADL, modified Schwab and England Activities of Daily Living Scale; K-MBI, Korean Version of Modified Barthel Index. * *p* < 0.05 compared with patients with the off-state; ^†^ *p* < 0.05 compared with mSEADL during on-state.

**Table 3:** Correlation coefficients between the novel-developed disability measurement and other disability measurements.

	Total Patients (*n* = 47)	According to the On–Off Phenomenon	
		Patients without the Off-State (*n* = 23)	Patients with the Off-State (*n* = 24)
Reference mSEADL	0.960 *	0.976 *	0.955 *
K-MBI	0.808 *	0.688 *	0.799 *
UDPRS Part II	−0.708 *	−0.705 *	−0.624 *

mSEADL, modified Schwab and England Activities of Daily Living Scale; K-MBI, Korean Version of Modified Barthel Index; UPDRS, Unified Parkinson’s Disease Rating Scale. * *p* < 0.01, Spearman’s correlation analysis.

## Data Availability

The data that support the findings of this study are available from the corresponding author upon reasonable request.
